# Antibody–Drug Conjugate Revolution in Breast Cancer: The Road Ahead

**DOI:** 10.1007/s11864-023-01072-5

**Published:** 2023-03-25

**Authors:** Thomas Grinda, Elie Rassy, Barbara Pistilli

**Affiliations:** Department of Cancer Medicine, Gustave Roussy, 114 Rue Edouard Vaillant, 94800 Villejuif, France

**Keywords:** Breast cancer, Antibody–drug conjugate

## Abstract

Antibody drug-conjugates (ADCs) have revolutionized the treatment of many types of cancer, including breast cancer. Recently, two new ADCs have been approved, trastuzumab deruxtecan and sacituzumab govitecan; both have demonstrated impressive improvements in overall survival, trastuzumab deruxtecan in all three subtypes of metastatic breast cancer and sacituzumab govitecan in luminal and triple negative metastatic breast cancer. These drugs are the results of significant progress and innovation in the construction of the three components of an ADC, the monoclonal antibody, the payload, and the linker, and of the discovery of new target antigens. ADC engineering has profoundly changed the paradigm of cancer treatment, on one side being effective on tumors considered inherently resistant to the payload class of drugs and on the other side demonstrating activity in tumors with very low target expression. Yet, it is likely that we are just at the beginning of a new era as the identification of new targets and the introduction of new ADC constructs and combinations will expand the field of ADC rapidly over the coming years.

## Introduction

Over the past decade, the rapid evolution of hybridoma technology, along with the development of more hydrophilic and blood-stable linkers and the introduction of a wider spectrum of highly potent payloads have led to the development of a third generation of antibody–drug conjugates (ADCs) presenting several advantages over traditional therapies [1–4]. Given the complex interactions of each component with the tumor and the tumor microenvironment, the activity of the new generation of ADCs goes beyond the selective tumor delivery of high cytotoxic payloads and expands to tumors that are considered inherently resistant to the payload class of therapeutics. Indeed, the antibody backbone maintains its own functions of tumor target modulation, since the linker conjugation does not affect the antigen-binding site, and of immune effector, being able to activate antibody-dependent cellular cytotoxicity (ADCC), antibody-dependent cellular phagocytosis, and complement-dependent cytotoxicity (CDC) through its Fc region [5]. Furthermore, the growing use of cleavable linkers allows the diffusion of the lipophilic payloads across the cell membrane with the possibility of killing the neighboring cells not expressing the ADC target, the so-called bystander effect [6]. Finally, the rapid development of new conjugation technologies and the growing use of hydrophilic linkers have led to more homogenous and stable ADCs, with a higher drug to antibody ratio and a lower immunogenicity [3]. Currently, 14 ADCs have obtained approval across different countries for solid tumors and hematologic malignancies, 3 of them are approved for the treatment of early or metastatic breast cancer (BC), upon demonstration of impressive improvements in overall survival (OS) among all three BC subtypes [7–12]. However, we are just at the beginning of a new era, it is likely that the ADC landscape will be rapidly enriched with new promising compounds based on novel technologies and target antigens. Unfortunately, ADCs have also resulted in new toxicities, that can take their toll on the patients’ quality of life and requires education for their optimal management [9, 13]. Currently, a plethora of trials is evaluating new ADCs and ADC-based combinations to further improve the efficacy outcomes and to better understand unidentified resistance mechanisms, specific toxicity, and lack of predictive biomarkers. In this review, we discuss the road ahead of ADCs in BC.

## Currently approved ADCs in breast cancer

### Targeting HER2

Ado-trastuzumab emtansine (T-DM1), a second-generation anti-HER2 ADC, was the first ADC to be approved for the treatment of patients with HER2-positive BC [14]. T-DM1, a trastuzumab-based ADC bearing a cytotoxic microtubule inhibitor as payload, through a thioether uncleavable linker significantly improved progression-free survival (PFS) and OS over standard therapy, in patients with HER2-positive BC treated in 2^nd^ line and beyond. In the phase III trial TH3RESA, 606 heavily pretreated (median of 4 lines) patients were randomized to T-DM1 (n = 404) or the treatment of physician choice (n = 198) [15]. The median OS was significantly longer in the experimental arm (22.7 vs 15.8 months; HR 0.68, 95%CI 0.54–0.85), despite 47% of the patients in the control arm received T-DM1 at progression. In the phase III EMILIA trial T-DM1 was compared to lapatinib plus capecitabine, the most used treatment at that time in 2^nd^ line, in 991 patients with HER2-positive BC previously treated with trastuzumab and taxanes [8, 16]. T-DM1 outperformed standard therapy with a substantial improvement of objective response rate (ORR, 43.6% vs 30.8%), median PFS (9.6 vs 6.4 months; HR 0.65, 95%CI 0.55–0.77) and OS (29.9 vs 25.9 months; HR 0.75, 95%CI 0.64–0.88) [16]. Given the maytansinoid payload, the most frequent adverse events (AEs) with T-DM1 were thrombocytopenia (grade 3, 14%) and liver toxicity (grade 3, 5%). Relying on the positive results in metastatic setting, T-DM1 was tested as adjuvant therapy in patients with residual invasive disease after neoadjuvant chemotherapy for HER2-positive BC. The practice-changing KATHERINE trial showed that adjuvant T-DM1 halved the risk of recurrences in comparison to adjuvant trastuzumab [7].

More recently, a second anti-HER2 ADC, trastuzumab-deruxtecan (T-DXd) has revolutionized the treatment of metastatic breast cancer. T-DXd is composed of a cleavable tetrapeptide linker and a payload derivative of exatecan, a camptothecin analogues, that inhibits topoisomerase I (TOPO1), leading to DNA breaks [9]. After the striking results of the phase II single-group DESTINY-Breast01 study that led to FDA accelerated approval in December 2019, further studies confirmed the greater efficacy of T-DXd over standard therapy in patients with HER2-positive and HER2-low (immunohistochemistry 1 + or 2 + and negative results on in situ hybridization) [9]. The pivotal phase 3 DESTINY-Breast03 trial comparing T-DXd and T-DM1 in metastatic HER2-positive BC randomized 524 patients who had previously been treated with a taxane and trastuzumab. The median PFS was 4 times longer with T-DXd as compared to T-DM1 (28.8 vs 6.8 months; HR 0.33, 95% CI 0.26–0.43). At a median study follow-up of 28.4 months (range, 0.0–46.9 months), median OS was not achieved in both groups, but the difference was significant (HR 0.64, 95% CI 0.47–0.87) [10]. Furthermore, the phase III DESTINY-Breast02 study that compared T-DXd to treatment of physician choice in patients with HER2-positive metastatic BC who have received prior T-DM1, confirmed the greater efficacy of T-DXd also in later lines of treatment, with a median PFS of 17.8 months compared to 6.9 months with standard of care (HR 0.36, 95% CI 0.28–0.45).

Relying upon preclinical evidence on the T-DXd bystander effect and the preliminary data from phase I, the Phase III DESTINY-Breast04 trial evaluated the efficacy of T-DXd in pre-treated patients with HER2-low metastatic BC who had received one or two lines of chemotherapy. A total of 557 patients were randomized to receive T-DXd or physician-selected chemotherapy. In the subgroup of patients with hormone receptor (HR)-positive (n = 494), T-DXd improved the median PFS (10.1 vs 5.4 months; HR 0.51, 95%CI 0.40–0.64) and OS (23.9 vs 17.5 months; HR 0.64, 95%CI 0.48–0.86). These benefits were also seen in patients with HR-negative BC, with a median PFS of 9.9 ( vs 5.1 months; HR 0.50, 95%CI 0.40–0.63) and OS of 23.4 (vs 16.8 months; HR 0.64, 95%CI 0.49–0.84), though with the limits of an exploratory analysis of 58 patients [17]. Interestingly, T-DXd showed activity also in patients with HER2-negative BC, as demonstrated in the phase II DAISY study, in which patients with metastatic BC were divided into three cohorts based on HER2 expression to receive T-DXd. Among patients enrolled in the high HER2 expression cohort (immunohistochemistry [IHC] 3 + or IHC2 + /ISH +), the ORR was 69.1% and the median PFS was 11.1 months. Patients in the HER2-low-expression cohort (IHC1 + or IHC2 + /ISH- tumors) had an ORR of 33.3% and a median PFS of 6.7 months. In the HER2-non-expressing cohort (IHC 0), the ORR was 30.6% and the median PFS was 4.2 months [18]. Overall, T-DXd had a generally manageable safety profile, with hematologic, low-grade gastrointestinal AEs being the most common. Interstitial lung toxicity (ILD) is the most concerning AE. In a pooled analysis of nine phase I and II T-DXd monotherapy, the overall incidence of ILD was 15.4%, most of which was low grade; 77.4% was grade 1 or 2, and in rare cases was lethal (grade 5, 2.2%). Factors potentially associated with an increased risk of ILD were age < 65 years, recruitment in Japan, T-DXd dose > 6.4 mg/kg, oxygen saturation < 95%, moderate/severe renal insufficiency, presence of pulmonary comorbidities, and time since initial diagnosis > 4 years [19]. Although the ILD pathogenesis is still unclear and under investigation, it is likely that a target-independent uptake of T-DXd into alveolar macrophages could be the key mechanism [20].

### Targeting TROP-2

The trophoblast cell-surface antigen 2 (Trop-2) is a transmembrane glycoprotein that acts as a calcium signal transducer. Present at low levels in normal tissue but overexpressed in epithelial tumors, including 80% of patients with BC [21], Trop-2 overexpression has been associated to tumor invasiveness and growth, as a result of the mesenchymal-to-epithelial transcription regulation [22]. Sacituzumab govitecan is a first-in-class ADC directed against Trop-2, in which the anti-Trop-2 antibody is coupled via a hydrolysable linker to SN-38, the active metabolite of the topoisomerase I inhibitor, irinotecan [23]. In the ASCENT trial, in patients with metastatic triple negative breast cancer (TNBC) who had received at least two prior systemic therapies, sacituzumab govitecan demonstrated, compared with physician-selected single-agent chemotherapy, an increase in median PFS (5.6 months versus 1.7 months; HR 0.41, 95% CI 0.32–0.52), OS (12.1 months vs 6.7 months (HR 0.48, 95% CI 0.38–0.59), and ORR (35% vs 5%) [11]. In the TROPiCS-02 trial, in patients with metastatic HR-positive BC who had received at least one endocrine-based therapy, including a prior CDK4/6 inhibitor, and 2 to 4 lines of chemotherapy for metastatic disease, sacituzumab govitecan was compared with investigator's choice. In the interim analysis, sacituzumab govitecan showed a statistically significant PFS improvement (5.5 vs 4.0 months: HR 0.66, 95% CI 0.53–0.83) and OS improvement (14.4 vs 11.2 months; HR 0.79, 95%CI 0.65–0.96, p = 0.02) over standard chemotherapy [24]. In the phase III ASCENT and TROPiCS-02 trials, the most clinically relevant grade ≥ 3 AEs were neutropenia, diarrhea and alopecia, primarily associated with SN-38 [11, 12]. While pharmacogenomic screening is not required when prescribing sacituzumab govitecan, severe neutropenia was doubled in patients homozygous for the UGT1A1*28 allele in the ASCENT trial [25].

## Future ADCs in breast cancer

Over the past decade, we have witnessed an unprecedented fast-paced expansion of ADCs and a substantial number of novel ADC constructs have entered the clinical development, leveraging new antibody backbones, often targeting multiple antigens or different epitopes, new payload concepts that include also non-chemotherapeutic agents, and a broader range of suitable targets, not only in the tumor but also in the tumor microenvironment. Here below, we summarize the main properties of novel ADC constructs, that are currently being explored in BC, with a particular focus on further potential clinical development (Table 1).

**Table 1:** Ongoing Clinical Trials Evaluating New Antibody–Drug Conjugates in breast cancer in monotherapy

Target	Antibody drug conjugate	Payload	Linker	DAR	Trial	Population	Phase	Status
HER2	Vic-trastuzumab duocarmazine (SYD985)	Duocarmycin (DNA alkylator)	Cleavable	2.8	NCT04602117	HER-positive advanced solid tumors or HER2-low breast cancer	I	Recruiting
	Disitamab-Vedotin (RC48)	MMAE	Cleavable	4	NCT03262935	Metastatic Breast Cancer	III	Active, not recruiting
					NCT05331326	HER2) expressing breast cancer with abnormal activation of PAM pathway	II	Recruiting
					NCT04400695	Locally Advanced or Metastatic Breast Cancer With Low Expression of HER2	III	Recruiting
					NCT03052634	Advanced Breast Cancer With HER2 Positive or HER2 Low Expression	I-II	Active, not Recruiting
					NCT03500380	HER2-positive Locally Advanced or Metastatic Breast Cancer	II-III	Recruiting
	ARX788	AS269 (tubulin inhibitor)	noncleavable	1.9	NCT04829604	HER2-positive Metastatic Breast Cancer Patients who were previously treated with T-DXd	II	Active, not recruiting
					NCT03255070	Breast Neoplasms	I	Active, not recruiting
	MRG002	MMAE	cleavable vc-linker	-	NCT05263869	HER2-positive Breast Cancer With Liver Metastases	II	Recruiting
					NCT04924699	HER2-positive unresectable locally advanced or metastatic breast cancer	II	Recruiting
					NCT04742153	HER2-low locally advanced or metastatic BC	II	Recruiting
	A166	Duostatin 5 (Tubulin targeting agent)	Cleavable	2	NCT05311397	unresectable, locally advanced or metastatic HER2-expressing solid tumors	I	Recruiting
					NCT03602079	HER2-positive **Breast Cancer**	I-II	Active, not recruiting
	GQ1001	DM1 (Tubulin targeting agent)	Intelligent Ligase dependent	2	NCT04450732	HER2-positive **Breast Cancer**	I	Recruiting
TROP2	Datopotamab deruxtecan	Deruxtecan	tetrapeptide-based cleavable linker	8	NCT03401385 (TROPION-PanTumor01)	Advanced solid tumors	I-II	Recruiting
					NCT05104866 (TROPION-Breast01)	Inoperable or metastatic HR + /HER2 − breast cancer	III	Recruiting
					NCT05374512 (TROPION-Breast02)	Locally recurrent inoperable or metastatic TNBC who are not candidates for PD-1/ PD-L1 inhibitor therapy	III	Recruiting
	SKB264	Belotecan (topoisomerase I inhibitor)	Stable	7,4	NCT04152499	Locally advanced unresectable/metastatic solid tumors (TNBC)	I-II	Recruiting
HER3	Patritumab deruxtecan	exatecan derivative	Tetrapeptide based	8	NCT02980341	Refractory, advanced/ unresectable mBC	I-II	Active, not recruiting
					NCT04699630	Locally advanced BC or mBC	II	Recruiting
					NCT04965766	First-line HER3-high, HER2 − ,HR + unresectable locally advanced BC or mBC	II	Recruiting
					NCT04610528	HR + / HER2 − tumor with nonmetastatic primary invasive BC untreated and recently diagnosed	I	Recruiting
LIV-1	Ladiratuzumab vedotin	MMAE	cleavable dipeptide linker	4	NCT01969643	Metastatic **Breast Cancer**	I	Recruiting
Nectin-4	Enfortumab vedotin	MMAE	protease-cleavable	3.8	NCT04225117	Treatment refractory advanced solid tumors (HR + /HER2 − or TNBC)	II	Active, not recruiting
CEACAM5	Tusamitamab ravtansine (SAR408701)	maytansinoid agent DM4	Cleavable	3.8	NCT02187848	advanced solid tumors	I-II	Active, not recruiting
					NCT04659603	Metastatic, refractory, CAECAM-5–expressing breast or pancreatic cancer	II	Recruiting
Mesothelin	RC88	MMAE	cleavable	4	NCT04175847	Locally advanced or metastatic mesothelin-positive solid tumors including TNBC	I	Recruiting
cMet	TR1801-ADC	pyrrolobenzodiazepine	cleavable	2	NCT03859752	Solid Tumors Expressing c-Met	I	Active, not recruiting
	RC108	MMAE			NCT04617314	Metastatic refractory solid tumors that express c-Met	I	Recruiting
5 T4	SYD1875	Duocarmycin (amicrotubuleinhibitor)	cleavable	1.9	NCT04202705	Advanced Malignant Solid Tumors	I	Recruiting
	ASN004	Auristatin F hydroxypropylamide	cleavable	10–12	NCT04410224	Metastatic refractory solid tumors	I	Recruiting
FRα	MORAb-202 (farletuzumab ecteribulin)	Eribulin	cleavable	4	NCT04300556	Solid Tumors including TNBC	I-II	Recruiting
	AMT-151	-	-	-	NCT05498597	Advanced Solid Tumor including TNBC	I	Not yet recruiting
	PRO1184	exatecan, a topoisomerase 1 inhibitor	Cleavable	-	NCT05579366	advanced and/or metastatic solid tumors (TNBC, HR + and HER2 +)	I-II	Recruiting
ROR1	NBE-002	Anthracycline-derivative PNU-159682	Non-cleavable linker	-	NCT04441099	Locally advanced or metastatic solid malignant tumors (TNBC)	I-II	Recruiting
ROR2	CAB-ROR2-ADC (Ozuriftamab vedotin)	MMAE	cleavable	3–4	NCT03504488	Locally advanced unresectable or metastatic solid tumors (TNBC)	I-II	Recruiting
B7-H3	MGC018 (vobramitamab duocarmazine)	Duocarmazine	cleavable	2.7	NCT03729596	Locally advanced or metastatic solid tumors (TNBC)	I-II	Active, not recruiting
B7-H4	SGN-B7H4V	MMAE	Cleavable	-	NCT05194072	Advanced Solid Tumors	I-II	
	AZD8205	AZ’0133 (topoisomerase 1 inhibitor)	cleavable linker	-	NCT05123482	Advanced Solid Tumors	I-II	Recruiting
Globo H	OBI-999	MMAE	Cleavable	-	NTC04084366	Advanced Solid Tumors with high Globo H–expressing solid tumors (H score ≥ 100)	I	Recruiting
KAAG1	ADCT-901	pyrrolobenzodiazepine	Cleavable	-	NCT04972981	Advanced Solid Tumors	I	Recruiting
CD205/Ly75	OBT076	DM4	Cleavable	-	NCT04064359	Recurrent and/or Metastatic CD205 + Solid Tumors	I	Recruiting

### New ADC constructs targeting known antigens

Several ongoing trials have been evaluating new ADC constructs directed towards known antigens, namely HER2 and Trop-2, with the aim of improving treatment efficacy and tolerability.

#### HER2

At least 8 different ADCs targeting HER2 have recently entered the clinical development, showing different profile of activity and toxicity.

Vic-trastuzumab duocarmazine (SYD985) is composed of a DNA-alkylating agent payload attached to trastuzumab via a cleavable linker [26, 27]. After a phase I trial that showed a remarkable clinical activity in heavily pretreated patients with HER2-positive and HER2-low BC and led to a FDA fast track designation, SYD985 compared favorably with standard of care in the phase III TULIP trial. The study met its primary endpoint by increasing median PFS (7.0 vs 4.9 months; HR 0.64, 95%CI 0.49–0.84) and showing a trend for OS benefit (HR 0.83, 95%CI 0.62–1.09). The most common treatment-emergent AE was ocular toxicity, with any grade conjunctivitis and keratitis reported in 76% of patients and grade 3 or higher keratitis and conjunctivitis (5.6 vs 0.0%) in 12.2 and 5.6% of patients. Notably, ILD was reported in 7.6% (5.2% grade 1–2) of patients treated with Vic-trastuzumab duocarmazine, including two grade 5 patients. events [27]. Different mitigation measures, such as prophylactic use of eye drops are being evaluated.

Disitamab Vedotin (RC48) is composed of a novel humanized monoclonal antibody targeting HER2, hertuzumab, characterized by a higher affinity for HER2 and a more potent ADCC [28], and a monomethyl auristatin E (MMAE) payload, conjugated by a cleavable linker [29]. In the dose escalating C001 CANCER phase I trial RC48 showed activity across patients with different level of HER2 expression. In the HER2-positive subgroup, RC48 1.5, 2.0, and 2.5 mg/kg doses yielded an ORR of 22.2%, 42.9%, and 40.0% and median PFS of 4.0, 5.7 and 6.3 months, respectively. In the HER2-low expressing subgroup, the ORR and median PFS were 39.6% and 5.7 months, respectively with RC48 2.0 mg/kg [30]. This compound has also obtained conditional approval by the National Medical Products Administration (NMPA) of China for the treatment of patients with HER2-positive locally advanced or metastatic gastric cancer and of patients with metastatic urothelial cancer.

ARX788 is an ADC based on a site-specific conjugation technology through a non-natural aminoacid, thus presenting high homogeneity. It is composed of a humanized HER2 antibody combined to amberstatin (AS269), a tubulin inhibitor. In a phase I trial enrolling only patients with HER2-positive BC, the ORR was 65.5% and the median PFS was 17.0 months [31]. The most common grade 3–4 AEs were ocular AEs (5.7%) and pneumonitis (4.3%). The ongoing phase 2 ACE-Breast-03 (NCT04829604) study is evaluating ARX788 activity and safety in patients with metastatic HER2-positive BC who are resistant to T-DM1, T-DXd, and/or tucatinib-containing regimens. However, the development of ARX788 is currently on hold.

Two other ADCs in clinical development containing a MMAE payload have also showed promising activity in patients with HER2 + BC. With the first, ALT-P7, the ORR was 77% and median PFS 6.2 months [32] in a phase I trial enrolling 27 heavily pretreated patients with HER2-positive BC progressing on at least two prior anti-HER2 therapies. The most frequent AEs included myalgia (33.3%), fatigue (25.9%), sensory neuropathy (22.2%), alopecia (22.2%), pruritus (22.2%) and neutropenia (22.2%). The second, MRG002, demonstrated significant activity across patients with HER2-low BC, with an ORR 34.1% and 37.5% [33] in IHC 1 + and 2 + , respectively. The reported AEs were neutropenia (53.6%), aspartate aminotransferase and alanine aminotransferase increase (46.4 and 32.1%), and alopecia (39.3%).

#### TROP-2

Datopotamab deruxtecan (Dato-DXd) is a third generation ADC targeting Trop-2 and composed of a IgG1 mAb coupled with a topoisomerase-I inhibitor (MAAA-1181a, an exatecan derivative) via a tetrapeptide-based cleavable linker, with a drug to antibody ratio of ~ 4:1 [34]. Multiple phase I-III studies are currently underway with this compound as single agent or in combination with immune checkpoint inhibitors. In the TROPION-PanTumor01 (NCT03401385) an ongoing phase 1 multicohort study, a 32% ORR and 80% disease control rate (DCR) was observed in 44 heavily pretreated patients with TNBC. Notably, a tumor shrinkage was reported also in 9 out of 13 patients previously treated with a topo-I-targeting ADC [35, 36]. The most frequent any grade AEs were stomatitis (73%), nausea (66%), vomiting (39%), fatigue (34%), and alopecia (36%) nausea (66%) and stomatitis (55%); grade 3 stomatitis was reported in 11% of cases. In the cohort of patients pretreated for RH + /HER2- metastatic breast cancer, the ORR was 29% and the DCR was 85%. The most common AEs were stomatitis (80%), nausea (56%), fatigue (46%), and alopecia (37%) [37]. Two phase III trials are currently evaluating the efficacy of Dato-DXd as compared to standard of care as first line therapy in patients with advanced TNBC (NCT05374512) and as 2^nd^ or 3^rd^ line therapy in patient with advanced HR-positive BC (NCT05104866). Also, a phase III trial is evaluating Dato-DXd with or without durvalumab in patients with stage I to III TNBC with residual invasive disease after neoadjuvant systemic therapy (NCT05629585).

### Novel selected targets

#### HER3

HER3 is a transmembrane growth factor receptor, overexpressed in 50% of BCs. HER3 mediates resistance to EGFR, HER2, PI3K/AKT/mTOR directed therapies and endocrine therapy and its allosteric function is critical for HER2-amplified cancer growth [38–40]. HER3 overexpression has been associated to higher risk of cancer progression and worse prognosis across different tumor types, including BC [41]. Patritumab deruxtecan (U3-1402, HER3-DXd) is an ADC directed towards HER3 and composed of a human IgG1 monoclonal (mAb, patritumab) linked to a topoisomerase I inhibitor payload (MAAA-1181a, an exatecan derivative) via a tetrapeptide based cleavable linker, with a high drug to antibody ratio of about 8 [42]. The phase 1/2 study U3-1402-J101 study included patients with HER3 + (IHC 2 + or 3 +) heavily pretreated metastatic BC: 113 with luminal, 53 with TNBC and 14 with HER2-positive BC. The ORR was 30%, 23% and 43%, respectively. Interestingly, HER3-DXd activity was independent of HER3 expression [43]. Likewise, in the TOT-HER3 study, which tested the clinical and biological activity of a single dose of HER3-DXd in patients with treatment-naïve early luminal BC, the ORR was independent of baseline HER3 IHC and ERBB3 mRNA expression level [44]. Further phase II and III studies are underway in early and metastatic setting (NCT05569811, NCT04965766, NCT04699630).

#### LIV-1

LIV-1 is a transmembrane protein with metalloproteinase activity, belonging to a subfamily of ZIP (Zrt, Irt-like proteins) zinc transporters, with a heterogeneous expression across different normal tissues. Moderate/high LIV-1 expression has been found in about 90% of both HR-positive BC and TNBC [45] and it is associated with nodal and distant metastatic progression, consistently with its role in the epithelial-mesenchymal transition, through E-cadherin downregulation [46]. Ladiratuzumab vedotin (SGN-LIV1A) is an ADC composed of a humanized monoclonal antibody targeting LIV-1, bound through a protease-cleavable linker to a potent microtubule-disrupting agent, the monomethyl auristatin E (MMAE) payload, is a synthetic analogue of dolastatin 10, which determines G2/M phase cell arrest and ultimately apoptotic cell death.

A phase I open-label dose escalation and expansion study is actively recruiting heavily pretreated patients with metastatic LIV-1 positive TNBC or HR-positive endocrine-resistant BC (NCT01969643). An interim analysis of the 44 patients with TNBC included in the dose escalation and expansion cohorts showed ORR 32% and median PFS 11.3 weeks (95% CI 6.1–17.1 weeks), with the most common all-grade AEs being fatigue (59%), nausea (51%) and peripheral neuropathy (44%), while neutropenia (25%) and anemia (15%) were the most frequent grade 3 and 4 AEs. The established RP2D was 2.5 mg/kg every 3 weeks [47], however a weekly schedule is also being investigated to determine whether it widens the therapeutic window. In the adaptive platform trial I-SPY2, neoadjuvant therapy with 4 cycles of SNG-LIV1A 2.5 mg/kg every 3 weeks followed by 4 cycles of doxorucibicin + cyclophosphamide did not increase pathologic complete response rate as compared to standard arm [47]. Although normal apoptotic death is non-immunogenic, preclinical evidence demonstrated that SGN-LIV1 induced ER stress and release of immunogenic cell death (ICD) hallmarks such as ATP and HMGB1 in LIV-1 positive cells [48]. Relying upon these data, 2 ongoing studies are evaluating the combination of SGN-LIV1A with either pembrolizumab (NCT03310957) or atezolizumab (NCT03424005) in patients with TNBC as first or second line therapy, respectively, regardless of PD-L1 and LIV-1 expression.

#### Nectin-4

Nectin-4 is a transmembrane glycoprotein which is involved in cell proliferation, migration and adhesion. A moderate to strong staining has been reported in 60% of bladder and BCs, while its expression is low in normal tissues [49]. Enfortumab vedotin (EV) is a third generation ADC targeting nectin-4 and composed of a fully humanized IgG1 antibody coupled with a microtubule inhibitor (MMAE), via a protease-cleavable linker. In September 2021 it has obtained FDA approval for the treatment of patients with metastatic urothelial cancer [50]. Currently, a multicohort, phase 2 study (NCT04225117) is assessing EV activity and toxicity in patients with different locally advanced solid tumors, including patients with pretreated HR-positive BC and TNBC. Nectin-4 expression is not a study inclusion criterion and is evaluated as exploratory biomarker [51].

#### CEACAM5

Another emerging target is the carcinoembryonic antigen (CEA)-related cell adhesion molecule 5 (CEACAM5), a transmembrane glycoprotein, which presents high expression across multiple tumor types including gastrointestinal, lung, and breast, where it is involved in tumor invasion and metastasis, while its expression is low in normal tissues [52]. Tusamitamab ravtansine (SAR408701) is a new ADC targeting CEACAM5. It contains a humanized monoclonal antibody coupled to DM4, a maytansinoid payload by a cleavable Nsuccinimidyl 4-(2-pyridyldithio) butyrate (SPDB) linker. In the phase 1 study, 92 patients with different level of CEACAM 5 expression were treated with escalating doses of SAR-408701, including 1 patient with BC. Overall, 2 partial responses were observed (ORR 7.1%) in the low expression and 13 PRs (ORR 20.3%) in the high expression cohort. The most common treatment emergent AEs were asthenia, decreased appetite, keratopathy, and nausea, each of them observed in 25% of participants [53]. Both phase 2 and phase 3 clinical trials of SAR-408701 are currently ongoing in nonsmall cell lung cancer and other solid tumors (NCT04154956, NCT04659603, NCT04394624, NCT05071053, NCT04524689).

### New ADC constructs

#### Antibody Technology

The technology of the antibody part of ADCs is constantly evolving with the objective of optimizing cell internalization rates, tumor permeability and the recruitment of cells from the tumor's microenvironment.

Bispecific ADCs, i.e. targeting two different receptors on the same cancer cell, or biparatopic ADCs, i.e. targeting two different epitopes on the same receptor, are intended to improve targeting specificity, internalization but also to minimize toxicity to healthy tissues. MEDI4276 combines a biparatopic antibody targeting two non-overlapping epitopes on HER2 site-specific conjugation to a tubulysin-based microtubule inhibitor payload. By virtue of an increased internalization and lysosomal trafficking and degradation, it has shown strong antitumor potential in in vivo models of T-DM1 resistance or low HER2 expression [54]. In a Phase I trial of patients with advanced or metastatic HER2-positive BC pretreated with anti-HER2 drugs, MEDI4276 resulted in 1/47 complete response (0.5 mg/kg) and 2/47 partial responses (0.6 and 0.75 mg/kg) but intolerable toxicity primarily on increased liver function tests [55]. Another concept is to use a poorly internalized but specific antigen with a rapidly internalized antigen. In this way, the rapidly internalized antigen accelerates the uptake of the poorly internalized antigen. In this sense, a bispecific ADC targeting HER2, and the prolactin receptor (PRLR) kills more efficiently than a HER2 targeting ADC. In contrast to HER2, PRLR is rapidly and constitutively internalized, and PRLR levels on the cell surface are sufficient to ensure internalization. Non-covalent cross-binding of HER2 and PRLR to the cell surface, using a bispecific ADC that binds to both receptors, significantly improves internalization and cytotoxic potential [56].

Probody drug conjugates are another innovative technology for expanding therapeutic windows. They are a new class of proteolysis-activated recombinant antibody prodrugs that exploit the activity of tumor proteases to deliver their therapeutic effects to the tumor microenvironment rather than to healthy tissues expressing the same target. Targeting the tumor within the tumor environment can reduce adverse effects, thereby increasing safety and/or dose [57]. Recently, CX-2009, a probody drug conjugate directed against CD166 (ALCAM) and conjugated to DM4, was studied in a phase I trial involving 92 patients, 39 of whom had advanced BC. Partial responses were observed in 5 BC patients, digestive and dose-dependent ocular toxicity being the most frequent AEs [58]. Currently, the development of the CX-2009 is interrupted.

Another development perspective is to replace antibody backbones with small molecules. Small molecule-drug conjugates (SMDCs) are still in the early stages of development, but they may have the advantage of being less immunogenic, easier to synthesize, and have a high potential for penetration of solid tumors as well as anatomical barriers [59].

### New payloads

Immunostimulatory antibody conjugates (ISACs) use an immunostimulant such as Toll-like receptor (TLR) agonists, interferon stimulating gene agonists (STING) as a payload. These immunostimulants cannot be safely administered systemically, but by using a specific targeting antibody, they can be delivered to either the tumor cell or microenvironment cell. In a preclinical mouse model, BDC-1001 an ISAC comprising a dual TLR7/8 agonist conjugated to HER2-targeted antibodies elicited a localized immune response resulting in tumor reduction of myeloid and T cells and immunological memory [60]. A Phase 1/2 dose escalation/expansion study to evaluate BDC-1001 ± pembrolizumab in patients with HER2-expressing solid tumors whose disease has progressed on standard therapy is currently enrolling (NCT04278144). SBT6050 is also an ISAC composed of a TLR8 agonist conjugated to a monoclonal antibody directed against HER2. It has shown encouraging results in vitro and in vivo, particularly in combination with trastuzumab [61]. Other ADCs containing cytotoxic radioisotopes have shown clinical activity such as ibritumomab tiuxetan, an anti-CD20 antibody linked to iodine-131 against lymphoma [62]. However, to our knowledge, ADCs containing cytotoxic radioisotopes are not currently under development in BC.

## Future ADCs combination strategies in breast cancer

### ADCs and immune checkpoint inhibitors

A wealth of preclinical evidence support the combination of ADCs with immunotherapy. As first, most of ADCs induce ICD, which consists of cell surface exposure or dying cells release of damage associated molecular patterns (DAMPs). DAMPs are recognized by the immune system and eventually lead to the engulfment of tumor cells by antigen presenting cells and cross presentation to cytotoxic T cells [63–65]. Second, antibody backbones generally maintain their immune effector functions, being able of activating ADCC and CDC through their Fc portion [2, 5]. Finally, microtubule-inhibitor payloads (maytansinoids, dolastatins, auristatins) and topoisomerase-I inhibitor payloads are able to directly induce dendritic cell activation [66, 67]. Altogether, these evidence represent a strong rationale for combining ADCs and immunotherapy, a combination that could further augment the T cell infiltration within the tumor microenvironment [68–71].

The earliest trials combining immune checkpoint inhibitors to ADC in BC were conducted in patients with HER2-positive BC. The randomized phase II KATE2, enrolling 330 pretreated patients with HER2-positiveBC, has shown that the addition of atezolizumab to T-DM1 did not result in a statistically meaningful PFS and OS benefits neither in the intention to treat (HR 0.82, 95%CI 0.55–1.23 and HR 0.74, 95%CI 0.42–1.30, respectively) nor PD-L1-positive population (HR 0.60, 95%CI 0.32–1.11 and HR 0.55, 95%CI 0.22–1.38 respectively) [72]. Adverse events were more common in the combination arm, mainly involving thrombocytopenia (13 vs 4%), increased aspartate aminotransferase (8 vs 3%) and anemia (5 vs 0%) and led to study termination due to futility. Pharmacokinetic and immunogenicity results were as expected with T-DM1 and atezolizumab monotherapy; notably anti-therapeutic antibodies for T-DM1 and atezolizumab were reported in 1.7% and 12.1% [72]. A smaller phase Ib trial (NCT03032107) evaluating the combination of T-DM1 and pembrolizumab among 20 pretreated patients with HER2-positive BC has reported an ORR of 20% (95% CI 5.7–43.7%) and median PFS of 9.6 months (95% CI 2.8–16 months). All treatment related AEs were grade 2 and 3 (65% and 20%, respectively). PDL-1 and tumor infiltrating lymphocytes did not correlate with response in this small cohort [73]. More recently, a phase 1b study (NCT03523572) has evaluated the combination of T-DXd and nivolumab in patients with heavily pretreated HER2-expressng (HER2-positive or HER2-low) metastatic breast and urothelial cancers [74]. The ORR was 65.6% (95% CI, 46.8–81.4) and 50% (95% CI, 24.7–75.3) and median PFS was 11.6 months (95% CI, 6.9-not reached) and 7.0 months (95% CI, 2.3–10.8), respectively [75]. These results were nevertheless disappointing in comparison to the efficacy outcomes of T-DXd monotherapy in patients with HER2-positive (ORR 60.9% and PFS 16.4 months in DESTINY-Breast02). The safety profile was as expected with each study drug monotherapy. Treatment related AEs occurred grade ≥ 3 were reported in 48.9%; ILD occurred in seven patients (14.6%) of whom 6 were grade 2 and 1 grade 5 [74]. In contrast, the combination of T-DXd and durvalumab showed promising results in the Phase Ib study, BEGONIA, a multi-arm trial evaluating different treatment combinations in the first-line treatment of metastatic BC. In the HR-/HER2 low BC cohort, ORR was 57% (26/46) and median PFS was 12.6 months (95% CI, 8-not met), independent of PDL1 status. The most common AEs across all grades were nausea (73%), fatigue (46%), and vomiting (30%). Five patients (9%) experienced treatment-related interstitial lung disease or pneumonia, mostly grade 1 or 2, and one grade 5 case associated with COVID [76].

The combination of ADC and immune checkpoint inhibitors was also evaluated in TNBC. The combination of ladiratuzumab vedotin and pembrolizumab was evaluated in the phase Ib trial (NCT03310957) enrolling 51 treatment-naïve patients with advanced TNBC [77]. In this ongoing trial, the 26 patients with a follow up of at least 3 months had an ORR of 54% (95% CI 33.4–73.4) that exceeds the efficacy of pembrolizmab monotherapy in the patient with PDL-1-positive TNBC (ORR 21.4% [95% CI 13.9–31.4] [78]. The most common grade ≥ grade 3 were neutropenia (16%), diarrhea, fatigue, hypokalemia, and maculo-papular rash (8% each), and abdominal pain, increased alanine aminotransferase, and urinary tract infection (6% each). Similarly, in the TNBC cohort of the BEGONIA trial, 47 patients receiving the combination of Dato-DXd, and durvalumab had an ORR of 79% (95% CI, 61%-91%) independent of PD-L1 expression. 100% of patients with a complete or partial response remained in response at 6-month follow-up. The most common AEs of any grade were gastrointestinal (nausea in 55% and stomatitis in 51%). There were no reported cases of interstitial lung disease/pneumonitis or thrombocytopenia [79].

Other combinations are being evaluated in phase I-II trials, such as: T-DXd with pembrolizumab (NCT04042701) or durvalumab or durvalumab plus paclitaxel (NCT04538742), in metastatic HER2-positive BC; T-DXd with durvalumab plus paclitaxel in metastatic HER2-low BC (NCT04556773); Sacituzumab govitecan with pembrolizumab (NCT04468061) or with atezolizumab (NCT03424005).

### ADCs and targeted therapies

The combination of targeted therapies and ADCs was primarily evaluated in HER2-positive BC. The combination T-DM1 and pertuzumab had shown synergistic activity in cell culture models and had an acceptable safety profile in a phase Ib/II study [80, 81]. In the MARIANNE trial enrolling pretreated patients with HER2-positive BC, the addition of pertuzumab to T-DM1 did not achieve a clinically significant OS benefit (51.8 vs 53.7 months) and the treatment related adverse event were similar between the two groups (48.6 vs 47.1%) [82]. The combination of T-DM1 and pertuzumab was also evaluated in the neoadjuvant setting in two trials [83, 84]. The KRISTINE study compared neoadjuvant T-DM1 plus pertuzumab with docetaxel, carboplatin, trastuzumab plus pertuzumab in 444 patients with stage II/III HER2 + BC. The combination of T-DM1 and pertuzumab resulted in shorter event-free survival than docetaxel-carboplatin-trastuzumab-pertuzumab (TCHP) therapy (HR 2.61, 95%CI 1.36–4.98) in relation to higher locoregional progression before surgery (6.7 vs. 0%) in T-DM1-pertuzumab-treated patients. In contrast, invasive disease-free survival after surgery was similar (HR 1.11, 95%CI 0.52–2.40) [83]. The pathological complete response rates with the T-DM1 arm was 44.4% compared to 55.7% with the standard regimen [83]. Similar results were achieved in the I-SPY phase II trial that randomized patients with clinical stage II/III HER2 + BC to T-DM1 plus pertuzumab, paclitaxel plus pertuzumab and trastuzumab, or paclitaxel plus trastuzumab followed by doxorubicin/cyclophosphamide, then surgery. The 3-year event free survival of the T-DM1 arm was 88% (95% CI 79–99%), compared to 92% (95% CI 84–100%) for paclitaxel plus pertuzumab and trastuzumab and 87% (95% CI 75–100%) for paclitaxel plus trastuzumab [84]. Notably, the degree of HER2 pathway signaling and phosphorylation at baseline are associated with response to the T-DM1 plus pertuzumab combination and can further identify highly responsive to this combination. In addition, the under-efficacy of T-DM1 combined with pertuzumab may be driven by intratumoral HER2 heterogeneity. Indeed, HER2 heterogeneity, defined as an area with ERBB2 amplification in > 5% but < 50% of tumor cells, or a HER2-negative area by FISH, showed complete response rates of 0% versus 55% in the non-heterogeneous subgroup with neoadjuvant therapy combining T-DM1 and pertuzumab [85].

Last, T-DM1 was combined to neratinib in the phase II NSABP Foundation Trial FB-10 [86]. Among the 27 patients with HER2 + BC enrolled in this study, 19 patients were evaluable for response and had an ORR of 63.2%. The translational research performed within this study suggested that resistance mechanisms to HER2 antibodies may be loss of HER2 receptor and high expression of p95HER2 [86].

The development of ADCs plus targeted therapy combinations in TNBC has also accelerated after the development of effective targeted therapies as well as ADCs in this setting. Poly ADP ribose polymerase (PARP) inhibitors, namely olaparib and talazoparib, approximately halved PFS in the OLYMPIAD and EMBRACA trials in patients with HER2-negative BC and *BRCA* germline mutations [87, 88]. The combination of rucaparib plus sacituzumab govitecan was reported in two cases of TNBC within the phase Ib SEASTAR Study [89]. The first patient had a deleterious homologous recombination repair gene (HRR) mutation and had a partial response and the second did not have an HRR mutation and had a stable disease at 24 weeks. The ongoing phase I/II trial NCT04039230 is evaluating the combination of sacituzumab govitecan plus talazoparib in patients with metastatic TNBC.

Several other phase I/II trials are ongoing and testing ADC-based combination with monoclonal antibodies, tyrosine kinase inhibitors, or endocrine therapies in the different subsets of BC.

### ADCs and chemotherapies

The first clinical evidence pointing out to the role of the ADC combination with chemotherapy came from the ECHELON-1 trial showing that brentuximab vedotin improved the outcomes of patients with advanced Hodgkin’s lymphoma [90]. Thereafter, the combination of ADC and chemotherapy was evaluated in solid tumors and exhibited a promising efficacy [91–93]. Few trials have addressed the combinations of chemotherapy and ADCs in BC. The phase I trial, STELA, showed an ORR of 85.7% among 14 evaluable patients treated with T-DM1 plus nab-paclitaxel and lapatinib. It is noteworthy that the pharmacokinetics of T-DM1 were not affected by the combination [94]. Ongoing basket trials, including patients with metastatic TNBC, are evaluating the combination of ADCs to chemotherapy (NCT02996825, NCT03102320, NCT04538742, NCT04556773).

All clinical trials exploring ACD in combination are summarized in Table 2.

**Table 2:** Summary of the ongoing trials evaluating ADC-based combinations in breast cancer

Antibody drug conjugate	Payload	Combined drug	Population	Trial	Phase	Status
Mirvetuximab sorvatansine (IMGN853)	DM4 (tubulin inhibitor)	Gemcitabine	Basket trial including mTNBC	NCT02996825	I	Active, not recruiting
Anetumab ravtansine	DM4	Gemcitabine, Cisplatine	Basket trial including mTNBC	NCT03102320	I	Completed
Ladiratuzumab vedotin (SGN-LIV1A)	MMAE (tubulin inhibitor)	Trastuzumab	Her2 + BC	NCT01969643	I	Recruiting
		Atezolizumab	mTNBC	NCT03424005	I/II	Recruiting
ARX788	ARX788	Pyrotinib	Her2 + BC	NCT04983121	I	Recruiting
Sacituzumab govitecan	SN-38 (topoisomerase 1 inhibitor)	Talazoparib	mTNBC	NCT04039230	I/II	Recruiting
		Alpelisib	HER2- BC	NCT05143229	I	Recruiting
		Atezolizumab	TNBC	NCT04434040	II	Recruiting
		Pembrolizumab	mTNBC	NCT05382286	III	Recruiting
		Pembrolizumab	TNBC	NCT04230109	II	Active not recruiting
Trastuzumab emtansine	DM-1	Abemaciclib	HER2 + BC	NCT04351230	II	Withdrawn^
		Utomilimab	HER2 + BC	NCT03364348	I	Completed*
Trastuzumab deruxtecan	Exatecan derivative (topoisomerase 1 inhibitor)	Pembrolizumab	Lung cancer and BC	NCT04042701	I	Recruiting
		Nivolumab	Urothelial cancer and BC	NCT03523572	I	Active not recruiting
		Tucatinib	HER2 + BC	NCT04539938	II	Recruiting
			HER2 + BC	NCT04538742	I/II	Recruiting
		Durvalumab		(module 1)		
		Pertuzumab		(module 2)		
		Paclitaxel		(module 3)		
		Paclitaxel + durvalumab		(module 4)		
		Tucatinib		(module 5, 6)		
			HER2-low BC	NCT04556773	I	Recruiting
		Capecitabine		(module 1)		
		Paclitaxel + durvalumab		(module 2)		
		Capivasertib		(module 3)		
		Anastrazole		(module 4)		
		Fulvestrant		(module 5)		
		Pertuzumab	HER2 + BC	NCT04784715		Recruiting

^There were no patients enrolled in this study

*Results not reported at the time of the review publication

BC: breast cancer; mTNBC: metastatic triple negative breast cancer; TNBC: triple negative breast cancer

## Conclusion and perspectives

Over the past years, we have witnessed a tremendous development of new ADCs in solid tumors, particularly in breast cancer, thanks to the availability of novel technologies for ADC production and the identification of new suitable targets. However, if on one hand the treatment landscape is being rapidly filled with new ADCs and novel ADC combinations, on the other hand we urgently need to understand ADC mechanism of action and resistance to better define future ADC sequential strategies in clinical practice. Mechanisms of resistance may involve the target antigens, ADC internalization, trafficking pathways, cell cycle modifications and signaling pathways, drug efflux pumps, lysosomal function, alteration of the target of the cytotoxic compound, and apoptotic dysregulation [95, 96]. Many of these resistance mechanisms are actively being evaluated in patients treated with third-generation ADCs. The translational research performed within the DAISY trial has shed lights on additional resistance mechanisms. Primary resistance may be associated to a high prevalence and spatial distribution of HER2 0 cells or to *ERBB2* hemizygous deletion and acquired resistance involved reduction in HER2 expression and occurrence of *SLX4* mutations [97, 98]. Similarly, resistance to sacituzumab govitecan was reported in 2 patients, of whom one lacked TROP2 expression and progressed rapidly, while a second one, who was a long-responder had initial high TROP-2 expression and focal amplification of *TACSTD2/TROP2* gene. At progression, analysis of distinct metastatic subclones of the latest patient showed acquired *TOP1 E418K* resistance mutation and subsequent frameshift *TOP1* mutation that conferred resistance to SN-38 and *TACSTD2/TROP2 T256R* missense mutation that determined a defective plasma membrane localization TROP-2 [99].

Undoubtedly, the more comprehensive knowledge of ADC mechanisms of resistance will allow in the coming future the selection of the most appropriate ADC sequential strategies for each patient according to the specific tumor characteristics upfront and the acquired resistance mutations. Being ADC modular, it is also likely that the development of ADC-platforms will furtherly evolve towards more personalized, patient-specific compounds in which the selection of the antibody backbone, the linker and the payload will be based on the specific tumor and tumor microenvironment activated pathways and expressing proteins.
