# Correction to: Antibody–Drug Conjugate Revolution in Breast Cancer: The Road Ahead

**DOI:** 10.1007/s11864-023-01092-1

**Published:** 2023-04-18

**Authors:** Thomas Grinda, Elie Rassy, Barbara Pistilli

**Affiliations:** Department of Cancer Medicine, Gustave Roussy, 114 Rue Edouard Vaillant, 94800 Villejuif, France

**Correction to: Current Treatment Options in Oncology**

10.1007/s11864-023-01072-5

The article "Antibody–Drug Conjugate Revolution in Breast Cancer: The Road Ahead", written by Thomas Grinda, Elie Rassy, and Barbara Pistilli, was originally published electronically on the publisher’s internet portal on March 25, 2023 without open access. With the author(s)’ decision to opt for Open Choice the copyright of the article changed on March 30, 2023 to © Authors 2023 and the article is forthwith distributed under a Creative Commons Attribution 4.0 International License, which permits use, sharing, adaptation, distribution and reproduction in any medium or format, as long as you give appropriate credit to the original author(s) and the source, provide a link to the Creative Commons licence, and indicate if changes were made.

The images or other third party material in this article are included in the article’s Creative Commons licence, unless indicated otherwise in a credit line to the material. If material is not included in the article’s Creative Commons licence and your intended use is not permitted by statutory regulation or exceeds the permitted use, you will need to obtain permission directly from the copyright holder.

To view a copy of this licence, visit http://creativecommons.org/licenses/by/4.0/.

